# Draft Genome Sequence of the Organophosphorus Compound-Degrading *Burkholderia zhejiangensis* Strain CEIB S4-3

**DOI:** 10.1128/genomeA.01323-14

**Published:** 2014-12-18

**Authors:** Armando Hernández-Mendoza, Fernando Martínez-Ocampo, Luis Fernando Lozano-Aguirre Beltrán, Elida Carolina Popoca-Ursino, Laura Ortiz-Hernández, Enrique Sánchez-Salinas, Edgar Dantán-González

**Affiliations:** a Departamento de Bioquímica y Biología Molecular, Facultad de Ciencias, Universidad Autónoma del Estado de Morelos, Cuernavaca, Morelos, Mexico; b Laboratorio de Investigaciones Ambientales, Centro de Investigación en Biotecnología, Universidad Autónoma del Estado de Morelos, Cuernavaca, Morelos, Mexico; c Programa de Genómica Evolutiva, Centro de Ciencias Genómicas, Universidad Nacional Autónoma de México, Cuernavaca, Morelos, Mexico

## Abstract

*Burkholderia* species are widely distributed in the environment. A *Burkholderia zhejiangensis* strain was isolated from pesticide-contaminated soil from an agricultural field in Mexico and identified as an organophosphorus compound-degrading bacterium. In this study, we report the draft genome sequence of *Burkholderia zhejiangensis* strain CEIB S4-3.

## GENOME ANNOUNCEMENT

Bacteria of the genus *Burkholderia* are broadly distributed in several ecological niches. Pesticides that are applied to agricultural soil control pests that disrupt crop production, but when pesticides persist and accumulate in soils, they can alter microbial processes. Methyl parathion (MP) is a toxic organophosphate insecticide that irreversibly inhibits acetylcholinesterase (1). The hydrolysis of MP generates *p*-nitrophenol (PNP) (2), a toxic environmental pollutant (3, 4). In 2012, Popoca-Ursino (5) reported three bacterial strains of the genus *Burkholderia* isolated from crop soils in Morelos, Mexico. Here, we present a bacterium identified as *Burkholderia zhejiangensis* strain CEIB S4-3 that has the capability to completely degrade PNP and use it as a carbon source. These observations indicate that this organism potentially has genes that simultaneously hydrolyze MP and degrade PNP.

Strain CEIB S4-3 was grown at 30°C with constant agitation for 12 h. Genomic DNA was obtained using the AxyPrep bacterial genomic DNA miniprep kit (Axygen). The concentration was calculated in a UV-Vis NanoDrop 2000 spectrophotometer (Thermo Scientific), and 5 µg of genomic DNA was sequenced in the Genome Analyzer IIX system (Illumina). We obtained a random data set of 34,641,784 paired-end reads 72 bases in length. Quality-based trimming was performed with a DynamicTrim (SolexaQA++) perl script, and genome assembly was accomplished using the SPAdes (version 3.1.1) program. The draft genome has 154 contigs, with a calculated 7,666,843 bp total length and an *N*_50_ contig size of 156,081 bp. We aligned the draft genome with 41 complete genomes (chromosomes and plasmids) from members of the genus *Burkholderia* using the NUCmer program. The draft genome has 86.95 and 87.6% identity and 4.57 and 5.31 Mb alignment coverage with *Burkholderia* sp. strain RPE64 (3 chromosomes, 2 plasmids, and 6,964,487 bp total length) (6) and *Burkholderia* sp. strain YI23 (3 chromosomes, 3 plasmids, and 8,896,411 bp total length) (7), respectively. This analysis suggests that *B. zhejiangensis* CEIB S4-3 has at least 2 chromosomes.

The contigs were analyzed on the RAST server, which identified 7,228 coding sequences (CDS). We identified the 16S rRNA gene of *B. zhejiangensis* CEIB S4-3 using the RNAmmer 1.2 server. We compared 101 16S rRNA genes from the genus *Burkholderia* and two 16S rRNA genes from the genus *Ralstonia* (outgroup). The sequences were aligned in the MUSCLE server, and a phylogenetic analysis was performed in the MEGA (version 6.1) program with a neighbor-joining algorithm using 1,000 replicates for bootstrapping.

The 7,228 predicted CDS were compared with the COG database, and 4,391 proteins (60.8%) were identified: 1,545 open reading frames (ORFs) (21.4%) had an identified function and 2,846 ORFs (39.4%) had an uncharacterized function. We also identified a methyl parathion-degrading (*mpd*) gene (99% identity with the MpdB protein from *Burkholderia cepacia*) (8) and two PNP catabolic gene clusters (*pnpABAʹE1E2FDC* and *pnpE1E2FDC*; identities ranging from 67 to 100% with PnpABE1E2FDC proteins from *Burkholderia* sp. strain SJ98) (9, 10). These catabolic genes will explain the capabilities of this bacterium to hydrolyze MP and degrade PNP completely.

### Nucleotide sequence accession number.

The draft genome sequence of *B. zhejiangensis* CEIB S4-3 (including 154 scaffolds) has been deposited in GenBank database under accession no. JSBM00000000.
